# Serological evidence of *Coxiella burnetii* as a causative agent of culture negative infective endocarditis

**DOI:** 10.1186/1471-2334-14-S3-P36

**Published:** 2014-05-27

**Authors:** Angelina Raghavendran, Venkatasan Naveen Kumar, Gopalakrishnan Sathya Narayanan, Thangam Menon

**Affiliations:** 1 Department of Microbiology, Dr. ALM PG Institute of Basic Medical Sciences, University of Madras, Taramani, Chennai, Tamil Nadu, India

## Background

Culture negative infective endocarditis (CNIE) accounts for 2.5 to 31% of all cases of infective endocarditis and is responsible for delay in diagnosis and treatment with profound impact on clinical outcome. *Coxiella burnetii* is one of the important etiological agents of culture negative infective endocarditis. The present study was undertaken to determine the role of *C burnetii* as an etiological agent in CNIE in South India.

## Methods

Out of a total of 124 patients with CNIE during the period from 2008 to 2012, 46 were selected for the present study and stored serum samples were analysed by a qualitative ELISA to detect the IgG antibodies against *C. burnetii* using ELISA Kit (NovaTec Immundiagnostica GmbH, Germany).

## Results

Among the 46 patients included in the study, 26 (57%) were male and 20 (43%) were female with mean age of 30 years in the range of 11-74 years. Out of the 46 patients screened for Phase I IgG class antibodies, 22 patients were found to be positive by ELISA. Among the 22 positive patients, majority of patients (90%) had native valve endocarditis predominantly of the mitral valve (60%). All the 22 patients presented with fever; about 64% of patients had rheumatic heart disease and 68% of them showed vegetation by echocardiography.

## Conclusions

Screening of all CNIE patients for *C. burnetii* Phase I IgG antibodies will help in initiating appropriate treatment and in bringing down the case fatality rates in culture negative infective endocarditis in India.

